# High Resource Overlap and a Consistently Generalised Pattern of Interactions in a Bat–Flower Network in a Seasonally Dry Landscape

**DOI:** 10.1002/ece3.70367

**Published:** 2024-10-09

**Authors:** Constance J. Tremlett, Mark Chapman, Kathryn H. Maher, Alexander Keller, Nico Blüthgen, Kelvin S.‐H. Peh, Veronica Zamora‐Gutierrez

**Affiliations:** ^1^ School of Biological Sciences University of Southampton Southampton UK; ^2^ Ecological Networks Lab, Department of Biology Technische Universität Darmstadt Darmstadt Germany; ^3^ NERC Biomolecular Analysis Facility, Department of Animal and Health Sciences University of Sheffield Sheffield UK; ^4^ Centre for Underutilised Crops University of Southampton Southampton UK; ^5^ Organismic and Cellular Networks, Faculty of Biology Biocenter, Ludwig‐Maximilians‐Universität München Planegg Germany; ^6^ CONAHCYT‐Centro Interdisciplinario de Investigación para el Desarrollo Integral Regional (CIIDIR) Unidad Durango Instituto Politécnico Nacional Durango Mexico

**Keywords:** *Anoura geoffroyi*, bat–flower interactions, *Choeronycteris mexicana*, *Leptonycteris nivalis*, *Leptonycteris yerbabuenae*, metabarcoding, Mexico, pollination networks

## Abstract

Pollination is an ecosystem process that is crucial to maintain biodiversity and ecosystem function. Bats are important pollinators in the tropics and are an integral part of complex plant–pollinator interaction networks. However, network analysis–based approaches are still scarce at the plant species and bat community levels. We used metabarcoding to identify plant taxa present in pollen from fur and faecal samples collected across 1 year from three nectar‐feeding bat roosts in central Mexico. We calculated the frequency of occurrence of plant taxa and assembled a zoocentric network of bat–plant interactions. We constructed a year‐long network, encompassing the entire period of sampling, two seasonal networks comprising the wet and dry seasons, and six individual networks from sampling at two‐month intervals across the year. Four species of nectar‐feeding bats interacted with 36 plant species from 16 families. We found highly generalised interaction patterns across networks corresponding with opportunistic feeding behaviour by bats, with little seasonal variation in network structure. There was high resource overlap between bat species, and bats visited a diverse range of plant species even during periods with a high abundance of particular resources in the landscape. The diverse diet of nectar‐feeding bats emphasises the importance of floristically rich natural habitats in the landscape to provide reliable foraging resources year‐round in a seasonally variable system. While a generalised network structure is thought to increase robustness, further research is necessary to understand how fluctuations in pollinator abundance and diversity in the face of land use and climate change may impact bat–flower networks and the consequences to plant communities.

## Graphical abstract

Bats are important pollinators in the tropics and are an integral part of complex plant–pollinator interaction networks. We used metabarcoding to identify plant taxa present in pollen from fur and faecal samples collected across 1 year from three nectar‐feeding bat roosts in central Mexico. We found highly generalised interaction patterns across networks corresponding with opportunistic feeding behaviour by bats, with little seasonal variation in network structure.

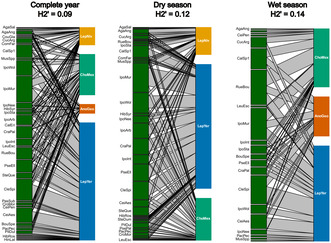

## 1 Introduction

Pollination is a crucial process for maintaining ecosystem function and biodiversity and is one of the most vulnerable stages to disturbance in the life cycle of plants (Neuschulz et al. 2016). Bats are important pollinators in the tropics and form an integral part of complex plant–pollinator dynamics (Fleming, Geiselman, and Kress 2009; Kunz et al. 2011). In Mexico, nectar‐feeding bats are keystone pollinators of much of the dominant vegetation in tropical forests and arid and semi‐arid zones, including columnar cacti (Cactaceae), paniculate agaves (Agavaceae) and canopy trees in the Malvaceae family (Fleming and Valiente‐Banuet 2002; Fleming, Geiselman, and Kress 2009). The study of plant–pollinator interactions allows for an increased understanding of community structure, with implications for ecosystem function and stability in the face of environmental change (Montoya, Pimm, and Solé 2006; Kaiser‐Bunbury and Blüthgen 2015; Zamora‐Gutierrez et al. 2021).

Across different ecosystems, plants generally have numerous potential pollinators, which can each visit various plants (Waser et al. 1996; Lucas et al. 2018). Generalised networks are thought to be more robust to fluctuations in pollinator diversity and abundance, allowing plant species to exchange one pollinator for another (Johnson and Steiner 2000). However, higher specialisation may be beneficial from the perspective of both the plant and pollinator (particularly when considering functional groups of pollinators rather than species), potentially increasing delivery of conspecific pollen to the plant (Armbruster 2014) and reducing interspecific competition for the pollinator (e.g., Maglianesi et al. 2014). Insect pollination networks typically show significantly higher specialisation than other mutualistic networks such as seed dispersal or ant–nectar networks (Blüthgen et al. 2007), while most hummingbird–plant networks across the Americas have moderate specialisation (Dalsgaard et al. 2011). Patterns of bat–flower network structure in the Neotropics vary according to abiotic variables and the bat species assemblage considered (Liévano‐Latorre, Varassin, and Zanata 2023; González‐Gutiérrez et al. 2022). However, studies of nectar‐feeding bat diets have revealed broad niches, even for morphologically specialised species (Gonzalez‐Terrazas et al. 2012; Diniz and Aguiar 2023a; Muchhala et al. 2024).

Patterns of generalism in a pollination network are dynamic, as diet breadths of pollinators can shift depending on environmental factors and resource availability (CaraDonna and Waser 2020). Seasonal differences in plant phenology and pollinator diversity and abundance can impact the properties of network structure (Burkle and Alarcón 2011; Souza et al. 2018). Evidence on the effect of resource availability and seasonality on pollination networks is variable. While some studies have found that a higher specialisation of flower visitors is facilitated by high resource availability (e.g., Venjakob et al. 2016), most have found that specialisation increases as resource availability decreases (e.g., Sperr et al. 2011; Tinoco et al. 2017; Souza et al. 2018; Sritongchuay, Hughes, and Bumrungsri 2019; de Oliveira et al. 2022; Stevens 2022), and this relationship can be influenced by multiple environmental factors driven by ecosystem seasonality. Across the seasonally dry tropical forests of the Caatinga, Brazil, bat–flower networks have a generalised pattern of interactions across seasons and years, with bat species showing high levels of interaction overlap (Cordero‐Schmidt et al. 2021). However, a meta‐analysis of 22 Neotropical bat–plant pollination networks found that higher seasonality (particularly in terms of precipitation) resulted in a lower niche overlap between bat species (Liévano‐Latorre, Varassin, and Zanata 2023). Functionally specialised species can switch seasonally to exploit different resources at different times of the year (Bender et al. 2017), and differences in the ability of nectar‐feeding bats to track lower density nectar sources (due to body size, flight characteristics and home range size) can impact interspecific competition and patterns of specialisation in networks (Tschapka 2004). Interaction patterns can therefore be flexible, influenced by multiple environmental and biological factors, leading to variation in network structure.

Studies on the diet of nectarivorous bats are extensive, and there is increasing interest in characterising bat–plant interactions through network analysis. However, network analysis–based approaches are still scarce at the plant species and bat community levels, with most studies addressing plant identification at coarse taxonomic resolution and focussing on a few interactions (but see Queiroz et al. 2021; Cordero‐Schmidt et al. 2021; González‐Gutiérrez et al. 2022; Diniz and Aguiar, 2023b). Reflecting on the importance of bats as pollinators across the Neotropics, network analysis, particularly at species level, is an important tool to help us identify key pollinator species and assess the vulnerability of plant–pollinator interactions to anthropogenic disturbance (Memmott, Waser, and Price 2004; Memmott et al. 2007; Sritongchuay, Hughes, and Bumrungsri 2019).

Here, we used metabarcoding to identify plant species in the year‐round diet of a nectarivorous bat community to characterise the seasonal dynamics of bat pollinator–plant interactions in a semiarid tropical landscape in central Mexico. This ecosystem harbours an extremely rich floristic (Banda et al. 2016) and nectarivorous bat diversity (Valiente‐Banuet et al. 1996), with a marked seasonality driven by well‐defined wet and dry seasons (Macías‐Rodríguez et al. 2018). However, compared with other studies conducted in seasonally dry landscapes, our study site is unique as it is located in one of the most important areas for the cultivation of an endemic columnar cactus named ‘pitayo’ (*Stenocereus queretaroensis* F.A.C Weber Buxbaum). Pitayo plants start flowering at the beginning of spring within the dry season, with fruits maturing into the early wet season. In this region, pitayos provide an unusually high availability of nectar, pollen and fruit that is not present in other seasonally dry landscapes.

We recorded the frequency of interactions between bats and plants, and assembled year‐long, seasonal and bimonthly bat–flower interaction networks. We calculated network indices to describe patterns of specialisation and the foraging behaviour of bat pollinators in all networks. We hypothesised that (1) there would be seasonal variation in network structure, due to likely differences in resource availability; (2) the overall network would be relatively generalist, owing to the broad diet of nectar‐feeding bats recorded by previous studies; and (3) pitayo would form the predominant part of the diet of all bat species during the flowering period, owing to the high availability of floral resources provided by pitayo plantations.

## 2 Materials and Methods

### 2.1 Study Area

Sampling was conducted at three bat roosts (Atoyac 19.99174, −103.50488; San Cayetano 20.13014, −103.5658; Cueva del Ermitaño 20.0812, −103.5965) in the Sayula Basin, Jalisco, in central Mexico. The Sayula Basin consists of a seasonal freshwater lagoon, framed by tropical deciduous forest (25% of total area; containing the highest floristic diversity), semiarid lowland areas with thorn scrub (2%), human settlements (7%) and agriculture (38%, Macías‐Rodríguez et al. 2018). The endemic cactus *Stenocereus queretaroensis* is an important regional crop that is mainly bat‐pollinated (Tremlett et al. 2020). The average annual rainfall is 660 mm, which mostly falls between June and October (around 65% of total annual rainfall occurs between June and August), with the dry season lasting from November to May (Pimienta‐Barrios, Pimienta‐Barrios, and Nobel 2004).

### 2.2 Sample Collection and DNA Extraction

We visited the three roosts every 2 months from April 2017 to February 2018, making a total of six sampling trips. We captured bats returning to the roost from feeding (numbers captured depended on activity) and collected samples of pollen from the head, chest and wings of captured bats using a cotton swab dabbed in 96% ethanol. The cotton swabs were subsequently placed in Eppendorf tubes of 1.5 mL with 96% ethanol. Bats were then placed individually in clean cotton bags for a maximum of 2 h to collect faecal samples. Faecal samples were placed in tubes with 96% ethanol, which was poured off after 24–36 h and replaced with fine silica gel following Nsubuga et al (2004). All samples were stored at −20°C until further processing in the laboratory.

We extracted DNA from the faecal samples using a modified CTAB method adapted from Doyle (1991), and from the pollen samples using an ammonium acetate method (Nicholls et al. 2000; Richardson et al. 2001). Further details are available in the Supporting Information.

### 2.3 PCR Amplification, Sequencing and DNA Reference Library

We used primer pair UniPlantF and UniPlantR to amplify part of the second internal transcribed spacer of nuclear ribosomal DNA (ITS2), a short region typically of 187–380 base pairs that provides a high taxonomic resolution (Chen et al. 2010; Moorhouse‐Gann et al. 2018; Table S1). All samples were processed in duplicate from the first PCR stage (after DNA extraction) resulting in two PCR replicates of each sample. Final pools contained 260 samples (including eight PCR negatives) and were sequenced on an Illumina MiSeq sequencing platform, using 250 bp paired‐end reads. Further details are available in the Supporting Information.

To improve taxonomic resolution, reference DNA sequences were generated and submitted to GenBank for some plant species that would potentially be visited by nectarivorous bats in the study region, which were selected after a literature review (Table S2; Supporting Information).

### 2.4 Bioinformatics

We processed the sequencing data for further analysis using VSEARCH v2.14.2 (Rognes et al. 2016) following the pipeline available at https://github.com/chiras/metabarcoding_pipeline (Leonhardt, Peters, and Keller 2022). Paired ends of forward and reverse reads were joined, and all reads shorter than 150 bp were removed. We then performed quality filtering (EE < 1) as described by Edgar and Flyvbjerg (2015) and *de‐novo* chimera filtering following UCHIME3 (Edgar 2016a). VSEARCH was used to define amplicon sequence variants (ASVs) (Edgar 2016b). By using VSEARCH against an ITS2 reference database for plant species of the sampled region, reads were directly mapped with global alignments with an identity cut‐off threshold of 97%. The reference database was compiled with the BCdatabaser (Keller et al. 2020) based on a list of plant species of Mexico (Villaseñor 2016) and then curated (Quaresma et al. 2023). To classify remaining reads still without taxonomic allocation at this point, SINTAX (Edgar 2016c) was used with a reference database comprising global plant species (Sickel et al. 2015; Quaresma et al. 2023).

Reads from the PCR negatives were then checked to provide a baseline for background contamination. The maximum number of reads from each plant species identified in negatives were subtracted from all other samples from the same plate (Drake et al. 2021). Negatives were then excluded from further analyses.

We converted read numbers to relative abundances to account for the variation in read depth both between samples and between sequencing runs, after excluding plant taxa from families not documented to have bat‐pollinated members (see below), to mitigate against the likely inclusion of pollen present in samples due to wind drift, pollen present on flowers due to dispersal from other pollinating agents such as birds or insects, or pollen accidentally inhaled or ingested by bats while grooming or drinking from nectar sources (families from Fleming, Geiselman, and Kress 2009; updated to reflect current taxonomic classification). We specified a minimum sequence percentage threshold of 1% to determine occurrences and retained for analysis plant taxa found in either replicate at above the 1% threshold (Deagle et al. 2018; Drake et al. 2021).

We used published databases of bat‐pollinated species (Fleming, Geiselman, and Kress 2009; Liévano‐Latorre, Varassin, and Zanata 2023) to generate a reference list of species and genera of plants that could be visited by bats for nectar or pollen (i.e., not fruit; Table S3). We excluded species from genera not included in this list. We then manually checked all remaining species to ensure that the taxonomic assignment was appropriate (i.e., the plant species has a geographical distribution within the study area), and the flower morphology has some characteristic associated with chiropterophily (i.e., time of flower opening, flower shape, size and colour, documented records of pollination syndrome), and excluded observations that did not meet these criteria (Supporting Information). We therefore took a cautious approach in including plants in the network, with the presence of pollen on the bodies of bats alone insufficient to assume floral visitation, as opposed to contamination due to the potential sources listed above.

We aggregated records of *Ceiba aesculifolia* and *C. acuminata* as we found it is not possible to distinguish between these species with the primers used. Pollen assigned to one taxon each in the *Agave, Bauhinia* and *Calliandra* genera were not classified to species owing to an inappropriate distribution of the species returned by the bioinformatics pipeline. These taxa are therefore shown on the bipartite network as *Agave sp. 1, Bauhinia sp. 1* and *Calliandra sp. 1* but are not included in analyses of network structure. All remaining taxa included in the network were classified at species level. Where we found documented flowering phenologies of plant species included in the network, we filtered results to keep occurrences of plant species only within their flowering season, to reduce the possibility of recording instances of frugivory in the bat–flower network (23% of occurrences were lost during this step; Table S3).

### 2.5 Network Analyses

We calculated the presence/absence of plant taxa in pollen and faecal samples collected from each bat individual in each month sampled and used these data to create a weighted adjacency matrix showing the summed interactions between bat species and plant taxa. Although occurrence‐based diet summaries can overestimate the importance of food items consumed in small quantities, we opted to use occurrence‐based metrics to avoid possible biases in DNA extraction, amplification and sequencing, and a lack of mock community data (Deagle et al. 2018), as well as potential differential digestion rates between pollen of different plant taxa (Herrera and Martinez Del Rio 1998).

We constructed a year‐long network, encompassing the entire period of sampling, two seasonal networks comprising the wet and dry season, and individual networks for each bimonthly sampling period. We calculated three network‐level metrics, focussing on quantitative indices, which have been found to be less sensitive to sampling intensity and network size:

1. Linkage density: represents the diversity of interactions per species, weighted by total interactions (Bersier, Banašek‐Richter, and Cattin 2002; Dormann et al. 2009) and computed as the average of the mean number of bat species visiting each plant species and the mean number of plant species visited by each bat species.
2. H2’: a quantitative index of network‐level complementary specialisation, which describes how strongly the interactions between bat–plant pairs differ from a random pattern where all bat species have the same preferences (Blüthgen, Menzel, and Blüthgen 2006). The expected minimum and maximum specialisation for the fixed diversity and abundance per species (marginal totals of observed network) defines the possible range for this index. Resulting values of H2’ range between 0 and 1, with values close to 1 indicating a highly specialised network (strongest flower species partitioning across bat species), and values close to 0 indicating a highly generalist network (highest overlap).
3. Niche overlap: mean similarity in interaction pattern between bat species, calculated using Horn's index. Values close to 0 indicate no common use of niches, while values close to 1 indicate complete niche overlap.

We tested values of quantitative metrics (H2’, linkage density and niche overlap) of constructed networks against 1000 iterations of a null model using a Patefield algorithm, which creates null models with marginal totals identical to those of the observed model (Dormann et al. 2009). Comparisons between observed networks and Patefield null models are recommended when considering network metrics sensitive to the abundance and diversity of interaction partners (Blüthgen and Staab 2024). All interaction network metrics were calculated using the ‘Bipartite’ package (Dormann et al. 2009) in R version 4.2.3 (R Core Team 2022), including only plant taxa identified to the species level in analyses.

### 2.6 Bat Foraging Behaviour and Resource Overlap

To assess the role of the bat pollinators within networks, we also calculated the discrimination/selectivity index d’ for each bat species, which measures how selective a flower visitor is relative to the abundance of available resources. The total number of interactions for each species is used as a measure of partner availability (Blüthgen, Menzel, and Blüthgen 2006). Values of d’ closer to 0 indicate an opportunistic flower visitor (i.e., the bat visits similar flowers to all other bat species), while those close to 1 indicate a highly selective flower visitor (i.e., the bat exhibits exclusive preferences for certain flowers). We compared values of d’ calculated for each bat species in each month in the wet season (*n* = 6) and each bat species in each month in the dry season (*n* = 8) with a Welch's *t*‐test.

We visualised resource overlap year‐round, and in the wet and dry seasons, using bat species as a predictor in a nonmetric multidimensional scaling ordination on a Bray–Curtis matrix, using the Vegan package (Oksanen et al. 2017). We plotted the scaling ordination on two dimensions. We then performed an ANOSIM to investigate whether seasonal resource use differed between bat species.

### 2.7 Sampling Completeness

We estimated the sampling completeness of bat–flower interactions in our constructed networks (year‐long, dry season and wet season) using the iNEXT package (Hsieh, Ma, and Chao 2016; Chao et al. 2014), considering each combination of bat‐plant interaction to be a ‘species’ and their frequency as ‘abundances’. We estimated the completeness of our sampling by dividing the observed interaction richness by the estimated richness. We also plotted individual‐based rarefaction and extrapolation curves with Hill numbers for each constructed network, using the same package (Figure S1).

## 3 Results

### 3.1 Nectar‐Feeding Bats

We captured a total of 233 nectar‐feeding bats throughout the year across the three roosts: 135 *Leptonycteris yerbabuenae* individuals, 49 *Choeronycteris mexicana*, 35 *Anoura geoffroyi* and 13 *L. nivalis. L. yerbabuenae* was present year‐round at Roost 1 with fluctuating abundance; low numbers of *C. mexicana* were present year‐round at Roost 2 except for during August; and Roost 3 was occupied by *Anoura geoffroyi* in June and October (wet season) and by *L. nivalis* in December and February (dry season). Across all bat species, we collected a total of 149 pollen samples from fur and 113 faecal samples for sequencing (we sequenced both faecal and pollen samples from 63 bats, faecal samples only from 23 bats, and pollen samples only from 76 bats). A higher proportion of captured *Leptonycteris yerbabuenae* bats carried pollen in the dry season, while there was no clear pattern for *Choeronycteris mexicana* (Table S4). *L. nivalis* and *A. geoffroyi* were each caught only in one season.

### 3.2 Bat–Plant Interactions

**FIGURE 1:**
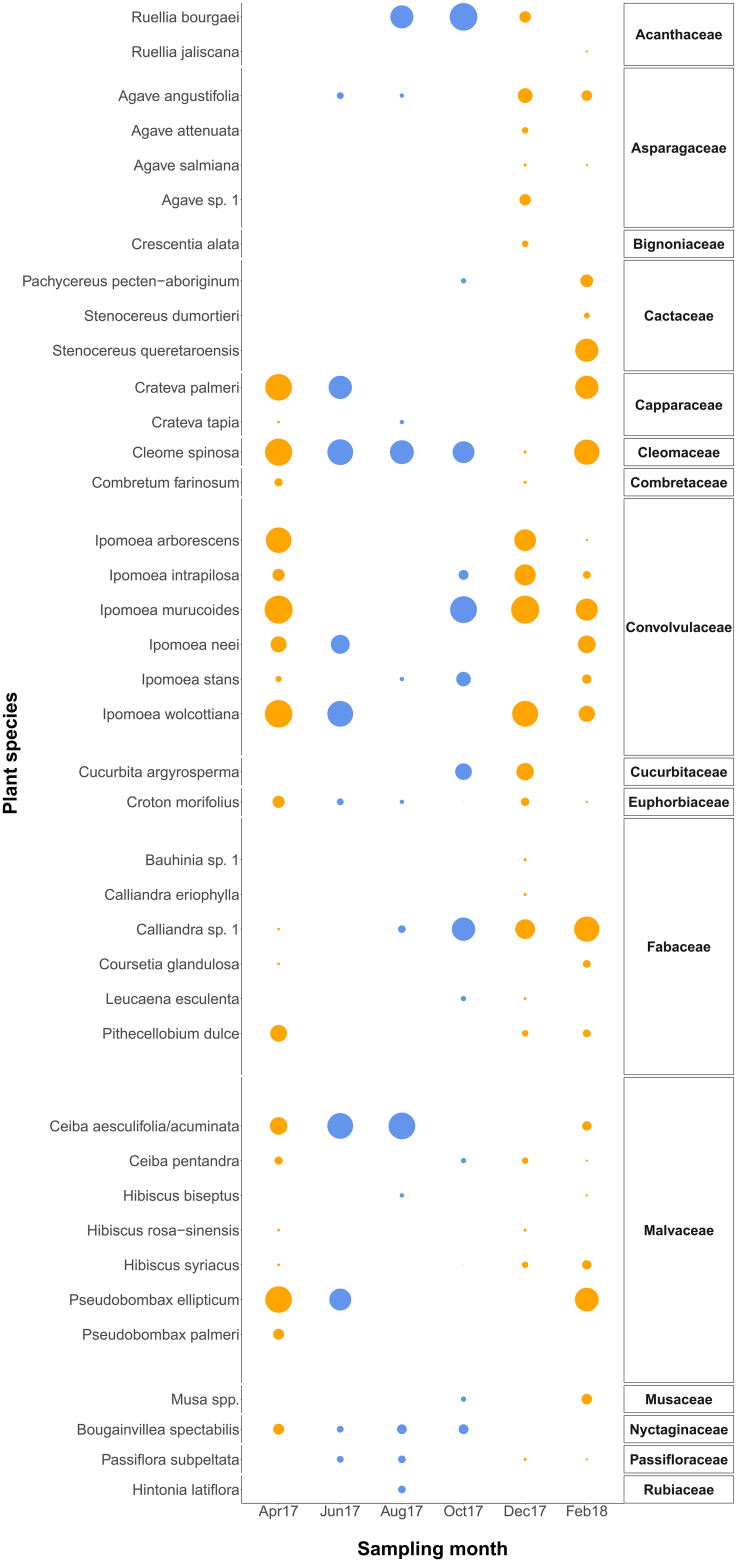
Per cent occurrence of plant species identified from pollen and faecal samples pooled across all bat individuals from all species for each sampling month. Occurences in the wet and dry seasons are shown in blue and orange, respectively.

We identified 36 plant species in pollen and faecal samples, from 16 plant families. Of these, 32 species were found in pollen samples while 27 species were found in faecal samples. Ten species were found in pollen samples only (*Agave salmiana, Cleome spinosa, Combretum farinosum, Cresecentia alata, Croton morifolius, Hibiscus rosa‐sinensis, Hintonia latiflora, Ipomoea stans, Pithecellobium dulce* and *Pseudobombax palmeri*) while four species were found only in faecal samples (*Calliandra eriophylla, Cucurbita argyrosperma, Leucaena esculenta* and *Ruellia jaliscana*). Fewer plant species were recorded in samples from the wet season compared with the dry season (a total of 20 and 35, respectively; Figure 1). We identified a mean 5.0 (±2.5 SD) plant species per bat sampled (pooled across bat species), and a mean of 3.8 (±1.8 SD) and 3.6 (±1.5 SD) plant genera and families, respectively.

Estimated sampling completeness of bat–plant interactions was highest when considering the complete year network than for the dry or wet season networks (Table 1; Figure S1).

**TABLE 1:** Observed and estimated (95% CL) species richness of plants visited by bat pollinators for the year and seasonal networks.

	Complete	Dry	Wet
*Leptonycteris yerbabuenae*			
Observed	34	32	16
Chao 1	36 (34–48)	43 (32–64)	17 (16–21)
Sampling completeness	94%	74%	94%
*Leptonycteris nivalis*			
Observed	21	21	—
Chao 1	26 (21–43)	26 (21–43)	—
Sampling completeness	81%	81%	—
*Choeronycteris mexicana*			
Observed	26	24	16
Chao 1	27 (26–35)	32 (24–61)	26 (16–54)
Sampling completeness	96%	75%	62%
*Anoura geoffroyi*			
Observed	10	—	10
Chao 1	11 (10–20)	—	11 (10–19)
Sampling completeness	91%	—	91%

### 3.3 Network Analyses

**FIGURE 2:**
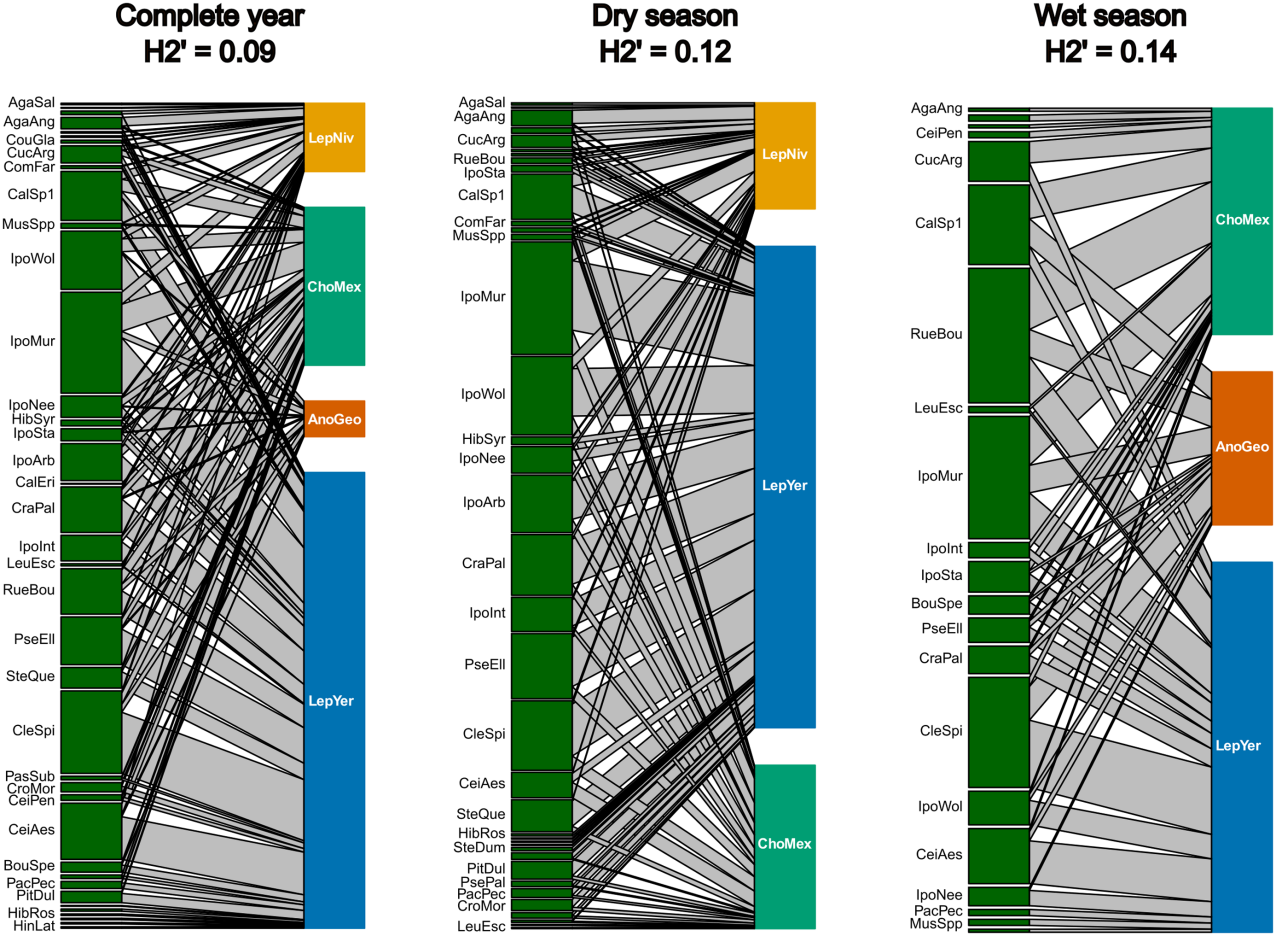
Bipartite interaction networks of plant species visted by nectar‐feeding bats in the Sayula Basin, Mexico, across the entire year, and during the wet (June to October) and dry season (December to April). Bat species labels correspond to: AnoGeo—*Anoura geoffroyi*, ChoMex—*Choeronycteris mexicana*, LepNiv—*Leptonycteris nivalis*, LepYer—*Leptonycteris yerbabuenae*. Plant species codes are defined in Table S6. The names of some infrequently encountered plants were removed from the plots for readability, but the interaction network data can be found in Supporting Information. Bipartite interaction plots for the bimonthly networks are shown in Figures S2, S3.

The complete network consisted of 802 occurrences of plant taxa in pollen and/or faeces sampled from bat individuals (Table S5). The three constructed networks showed a highly opportunistic distribution of bat species across plant species visited, with low values of H2’ (network‐level specialisation) and linkage density (diversity of interactions) and high values of niche overlap. Patterns of linkage density were significantly nonrandom (i.e., lower than expected if bats randomly interact with flowers) in the yearly, dry and two of the monthly networks, while H2’ was significantly non‐random (i.e., larger than expected if bats randomly interact with flowers) in all networks except April and June (Figure 2; Table 2). There was no difference in H2’ or niche overlap between the wet season months and the dry season months, while linkage density was lower in the wet season (Figure S4).

**TABLE 2:** Network metrics across the year‐round network, seasonal networks, and bimonthly networks (sample size too small to compute network metrics in August). Networks include plant species identified in pollen and/or faecal samples.

Network	No. samples	No. bat species	No. plant species	Linkage density	H_2_’	Niche overlap
Year	149	4	36	9.74***↓	0.09***↑	0.72***↓
Dry	88	3	35	9.05***↓	0.12***↑	0.79***↓
Wet	61	3	20	6.40^NS^↓	0.14**↑	0.77**↓
Apr	28	2	20	7.08^NS^	0.05^NS^	0.96^NS^
Jun	12	3	10	4.20^NS^	0.14^NS^	0.81^NS^
Aug	19	1	11	—	—	—
Oct	30	3	13	4.55^NS^	0.12*↑	0.82^NS^
Dec	28	3	20	5.33*↓	0.18*↑	0.77^NS^
Feb	32	3	24	6.97**↓	0.18*↑	0.66*↓

*Note:* Observed pattern ↑higher or ↓lower than random associations in null models.

*** *p* ≤ 0.001.

** *p* ≤ 0.01.

* *p* ≤ 0.05.

^NS^ Not significant.

### 3.4 Bat Foraging Behaviour and Resource Overlap

Low values of the discrimination/selectivity index d’ were observed throughout the year, with no difference between dry season and wet season months (Welch's *t*‐test: *t =* 0.28, *p* = 0.78; Table 3).

**TABLE 3:** Blüthgen's discrimination/selectivity index d’, calculated for flower‐visiting bat species.

	Complete	Dry	Wet	Apr	Jun	Oct	Dec	Feb
*Leptonycteris yerbabuenae*	0.06	0.08	0.12	0.05	0.16	0.08	0.18	0.16
*Choeronycteris mexicana*	0.04	0.06	0.20	0.05	0.25	0.13	0.15	0.25
*Anoura geoffroyi*	0.14	—	0.10	—	0.03	0.18	—	—
*Leptonycteris nivalis*	0.21	0.24	—	—	—	—	0.22	0.14

**FIGURE 3:**
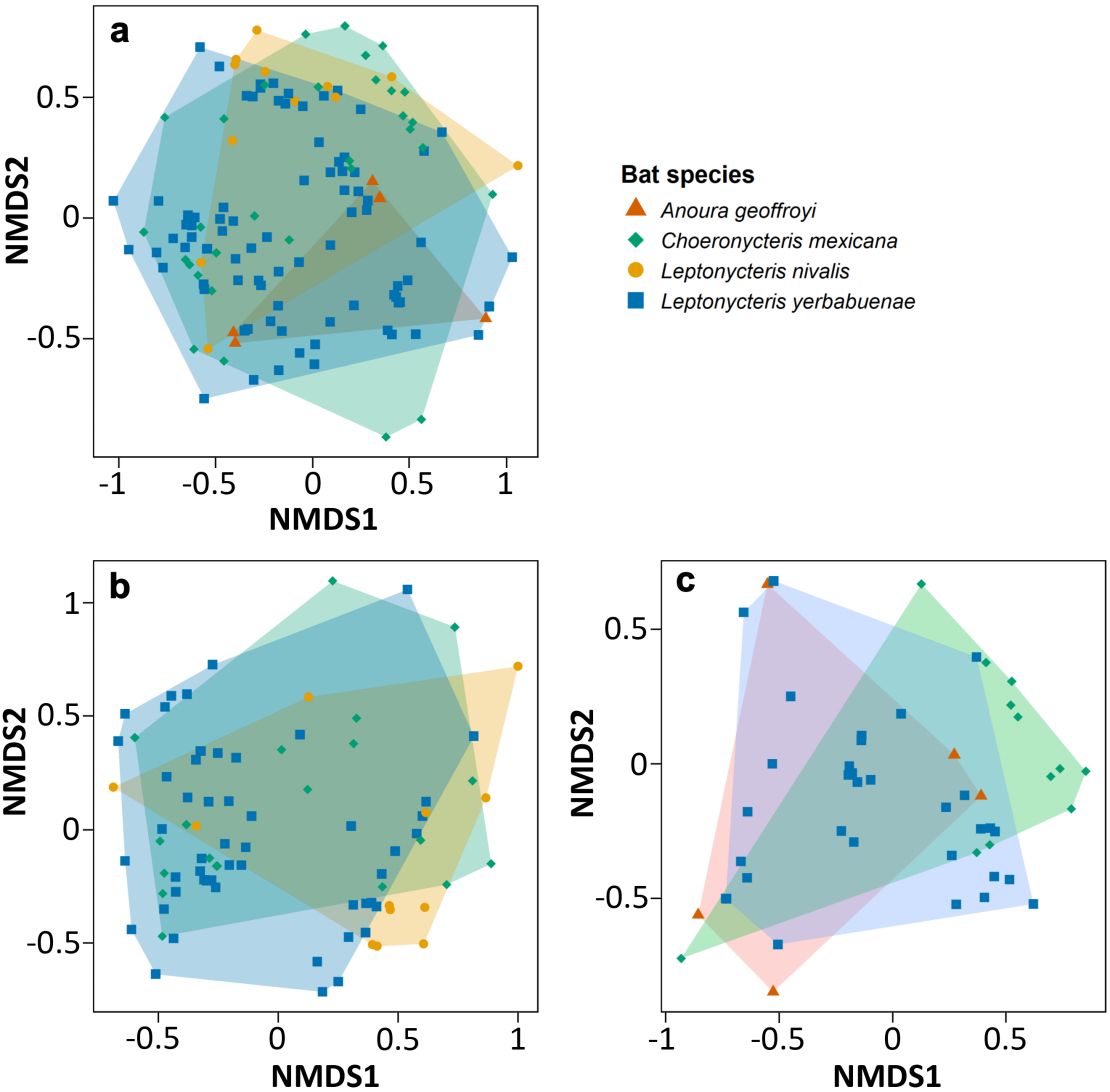
NMDS plots and convex hulls visualising distinctiveness of bat‐plant interactions between bat species: (a) across the whole year; (b) during the dry season months; and (c) during the wet season months.

Bat species was a small but significant predictor of resource use during the wet (*R* = 0.212, *p* < 0.001) and dry (*R* = 0.075, *p* < 0.05) seasons, but not when considering resources used across the entire year (*R* = −0.007, *p* = 0.56; Figure 3). The contribution of bat species to explain resource use was higher in the wet season.

## 4 Discussion

The year‐round bat‐flower network showed a highly generalised pattern of interactions, despite changes in bat species composition and differing resource overlap in the wet and dry seasons, indicating high overall network stability. Furthermore, low values of discrimination/selectivity (d’) for all bat species, low values of network specialisation (H2’) and high niche overlap suggest largely opportunistic feeding behaviour. Our results are consistent with other generalised interannual bat–flower networks in the Neotropics and corroborate the relatively high feeding plasticity of nectar‐feeding bats (Cordero‐Schmidt et al. 2021; Queiroz et al. 2021).

Network structure is influenced by various factors, including study design, the timing of resource availability, phylogenetic relationships between bat species and differences in foraging behaviour. Studies basing network metrics on direct observations of flower visits (e.g., Sritongchuay, Hughes, and Bumrungsri 2019) tend to report a higher specialisation and lower niche overlap than those identifying plant taxa from pollen samples, as visitation records are less effective at capturing the full range of plant resources used by the bat community (Bosch et al. 2009; Dorado et al. 2011).

Our network focussed on specialist nectarivorous bats sampled at the roost on return from foraging trips and included both pollen and faecal samples. We were therefore able to characterise a representative network of flowers visited by these nectarivorous bat species, removing any bias of sampling caused by choice of study sites or habitats. However, this approach also meant that facultative nectar‐feeding bats from other guilds (i.e., frugivores) were not included in our network (as these species did not roost communally with the specialist nectar‐feeding bats sampled and occur at lower abundances than our focal species), which could impact aspects of network structure in some temporal subsets of the network. For example, bat–flower networks were found to be more specialised in the Brazilian Cerrado, where frugivorous bats dominated the flower‐visiting niche in forests, and during times of very low fruit availability (Diniz and Aguiar 2023b). Thus, niches of flower‐visiting bats were driven not only by their ability to exploit certain flower types (i.e., morphology) but also by spatiotemporal overlap of resources and pollinators (Diniz and Aguiar 2023a). We found the lowest network specialisation in the month where only the two nectar‐feeding bat species with resident populations were present (in all other months an additional species was present).

Phylogenetic distance between specialist nectar‐feeding bat species may be a more important factor than morphological differences in determining resource partitioning: lower niche overlap was found in communities composed of species from different phylogenetic groups, unrelated to morphological traits (Liévano‐Latorre, Varassin, and Zanata 2023), while morphological specialisation appears to allow nectar‐feeding bats to access a wider range of floral resources without necessitating switching to other resources such as insects or fruit (Diniz and Aguiar 2023a; Gonzalez‐Terrazas et al. 2012; Muchhala et al. 2024). The low network specialisation and high niche overlap found in our study is likely influenced by the low phylogenetic distance between the four bat species included in our bat–plant network, which are all found within the obligatory nectar‐feeding subfamily Glossophaginae (with two species in the same genus; Rojas, Warsi, and Dávalos 2016). Modules of bat‐flower genus networks across central‐North America were found to be dominated by five bat genera including both specialist and facultative nectar‐feeders (*Anoura, Artibeus, Glossophaga, Hylonycteris* and *Leptonycteris*; González‐Gutiérrez et al. 2022). Specialist nectar‐feeders acted as hubs, interacting with a high number of plants. Of these modules, the *Leptonycteris* and *Anoura* genera are included within our network, which demonstrated strong associations with the Cactaceae and Asparagaceae, and Campanulaceae plant families, respectively.

We found little seasonal difference in network structure, despite seasonal fluctuations in floral resources and bat pollinator diversity and abundance in our system (Lobo et al. 2003; Borchert et al. 2004). A higher seasonality in precipitation is associated with lower niche overlap, likely due to influences on floral resource availability (Liévano‐Latorre, Varassin, and Zanata 2023). The abundance of nectar‐feeding bats at the roosts was highest in the dry season, when we also identified a higher number of plant taxa in pollen and faecal samples, and captured a higher percentage of bats carrying pollen, all presumably indicating a higher floral resource availability during this time. The abundance of nectar‐feeding bats is highly correlated with food availability, as bat populations time their reproductive activity and local and long‐distance migrations to synchronise with peak resource availability (Heideman and Utzurrum 2003; Stoner et al. 2003; Peñalba, Molina‐Freaner, and Rodríguez 2006). A higher availability of floral resources during the dry season and the large foraging ranges of bats may allow the convergence on favoured resources and reduce competition (Fontaine, Collin, and Dajoz 2008; Tinoco et al. 2017; Stevens 2022). However, though we found (slightly) greater resource overlap in the dry season, consistent with some previous studies of nectar‐feeding bats (Sperr et al. 2011; Sritongchuay, Hughes, and Bumrungsri 2019), both the dry and wet seasons showed a highly generalised pattern of bat–flower interactions. Nevertheless, the inclusion of facultative nectar‐feeding frugivore species in the network may impact patterns of specialisation between seasons, depending on the temporal availability of fruit (Diniz and Aguiar 2023a, 2023b).

Furthermore, nectar‐feeding bats in our study visited a high diversity of plants even during times of high abundance of particular resources in the landscape (flowering of monoculture plantations of the columnar cactus *Stenocereus queretaroensis*). Although *S. queretaroensis* appeared to be an important food resource, present in pollen and/or faecal samples from 22 of 32 bats captured in February, we found that 95% of the bat individuals that had visited *S. queretaroensis* had also visited other plant species, and furthermore that these individuals did not visit fewer plant species on average than bats in the same month that had not visited *S. queretaroensis*. In contrast, bats foraging in banana plantations in Costa Rica had a simplified, homogeneous diet compared to those foraging in forests (Alpízar, Schneider, and Tschapka 2020). Fine‐scale resource plant selection can be influenced by various factors determining foraging efficiency, including the density and spatial distribution of floral resources in the wider landscape, the quantity and sugar concentration of nectar rewards, flight costs, and inter‐ and intraspecies interactions with coexisting nectar‐feeding bats (Tschapka 2004). *S. queretaroensis* produces a high volume of hexose‐rich nectar (Ibarra‐Cerdeña, Iñiguez Dávalos, and Sánchez‐Cordero 2005), and the apparent nonlimitation of available resources during this time suggests that competitive interactions are unlikely to play a role in determining the foraging behaviour of the bats. We suggest that the concurrent high availability of other food plants (indeed, we observed the highest diversity of plants in the diet in February) during this time allows bats to forage opportunistically on their way to and from key foraging grounds from a diverse number of plant species. However, the use of presence/absence metrics may have underestimated the relative importance of *S. queretaroensis* in the diet, as all plant species are given equal weighting regardless of the proportion of the food intake they represent.

The occurrence of plant taxa in our samples indicated a pattern of sequential flowering of bat‐pollinated species throughout the year, and continuous or subannual flowering in some species, consistent with that observed in other bat‐pollinated flower assemblages (e.g., Heithaus, Fleming, and Opler 1975; Bullock and Solis‐Magallanes 1990; Sazima, Buzato, and Sazima 1999; Stoner et al. 2003; Cortés‐Flores et al. 2017). This provides a continuous supply of floral resources for bat pollinators and encourages the availability of bats as pollinating agents that can reside year‐round. *Leptonycteris* bats in tropical and sub‐tropical areas, including central Mexico, have been found to have a more diverse diet than seasonal populations in northern parts of their range, which feed primarily on Agavaceae and Cactaceae (Fleming and Nassar 2002). Our study emphasises the importance of maintaining heterogeneous natural habitats in the landscape to provide a diversity of resources for nectar‐feeding bats.

Generalised interactions within plant–pollinator networks have traditionally been viewed as competitive because of the cost to plants associated with the delivery of heterospecific, rather than conspecific, pollen (Morales and Traveset 2008; Flanagan et al. 2009; Ashman and Arceo‐Gómez 2013). However, there is increasing evidence that facilitation via pollinator‐sharing can be advantageous to plant communities (Tur et al. 2016; Aparecida Lopes et al. 2021), and generalist pollinators can be vital to meta‐network structure and resilience by linking subsets of the network and facilitating gene dispersal (González, Dalsgaard, and Olesen 2010). A high abundance of generalist pollinators can make an important contribution to pollen transport between conspecific plant individuals (Larsson 2005). Bat‐pollinated species often occur naturally at low densities and tend to be self‐incompatible and highly reliant on bats as mobile pollen dispersal agents (Herrerías‐Diego et al. 2006; Fleming, Geiselman, and Kress 2009; Quesada et al. 2013; Ratto et al. 2018). Morphological trait matching also influences pollination success, with diverse flower shapes and designs resulting in differential pollen placement across the body of bats, thus promoting conspecific pollen delivery (Muchhala 2008; Stewart and Dudash 2016a).

Bats are highly mobile, and the capacity for long‐distance travel is a particular characteristic of the bat species assemblage constituting our network, with three of the four species being migratory. *Leptonycteris yerbabuenae* individuals can travel up to 100 km per night during foraging trips (Goldshtein et al. 2020), while smaller glossophagines cover 50 km or less (Tschapka 2004; Rothenwöhrer, Becker, and Tschapka 2011). Bats in our network may therefore travel greater distances than those in other networks in search of resources, increasing the likelihood of encountering and opportunistically interacting with, a higher number of plant species (Stewart and Dudash 2016b). The high mobility of *Leptonycteris* bats also allows them to commute further to forage even when food resources are present nearer to the roost, perhaps to avoid competition and thus reduce niche partitioning (Ober, Steidl, and Dalton 2005). Additionally, while we found no resource partitioning of plant species, nectar‐feeding bat species may display territorial behaviour and partition individual plants or foraging areas (Lemke 1984; Goldshtein et al. 2020).

Higher generalisation in a plant–pollinator community should increase functional robustness and decrease vulnerability to changes in species diversity and abundance, for both plants and pollinators, as impacts are spread more evenly across the network (Kaiser‐Bunbury and Blüthgen 2015). Changes in climate and land‐use are projected to reduce numbers of potential bat–plant interactions in Mexico through changes in plant distributions, with increasing temperatures also predicted to cause a decrease in bat pollinator species richness, particularly in seasonally dry tropical forest (Zamora‐Gutierrez et al. 2021). Furthermore, anthropogenic impacts such as deforestation caused mainly by agricultural and cattle ranching activities are exerting a strong pressure on natural vegetation cover in the area (Macías‐Rodríguez et al. 2018). *Stenocereus queretaroensis* and other crop plantations are largely established in areas originally occupied by flower‐rich tropical dry forest, and the area under production is increasing yearly (Macías‐Rodríguez et al. 2018; SIAP 2018). Habitat loss or degradation can result in changes to bat foraging behaviour, which can lead to decreased visitation rates to flowers (Quesada et al. 2004), a more restricted diet (Alpízar, Schneider, and Tschapka 2020), and impact the delivery of conspecific pollen to flowers (Fuchs, Lobo, and Quesada 2003; Sritongchuay, Hughes, and Bumrungsri 2019), with possible implications for network structure.

## Author Contributions

**Constance J. Tremlett:** conceptualization (supporting), data curation (lead), formal analysis (lead), funding acquisition (supporting), investigation (equal), methodology (equal), project administration (equal), visualization (lead), writing – original draft (lead), writing – review and editing (equal). **Mark Chapman:** funding acquisition (supporting), methodology (supporting), resources (supporting), writing – review and editing (supporting). **Kathryn H. Maher:** methodology (supporting), resources (lead), writing – review and editing (supporting). **Alexander Keller:** data curation (supporting), methodology (supporting), resources (supporting), software (lead), writing – review and editing (supporting). **Nico Blüthgen:** methodology (supporting), writing – review and editing (supporting). **Kelvin S.‐H. Peh:** conceptualization (supporting), funding acquisition (equal), methodology (equal), project administration (equal), supervision (equal), writing – review and editing (supporting). **Veronica Zamora‐Gutierrez:** conceptualization (lead), funding acquisition (equal), investigation (equal), methodology (equal), project administration (equal), writing – original draft (supporting), writing – review and editing (equal).

## Conflicts of Interest

The authors declare no conflicts of interest.

## Statement on Inclusion

Our study brings together authors from a number of different countries, including scientists based in the country where the study was carried out. Whenever relevant, literature published by scientists from the region was cited; efforts were made to consider relevant work published in the local language.

## Supporting information


**Data S1.** Supporting information.

## Data Availability

Data available from the Dryad Digital Repository. Code for bioinformatic processing at https://github.com/chiras/metabarcoding_pipeline.
