# Supplementary material for: High Resource Overlap and a Consistently Generalised Pattern of Interactions in a Bat–Flower Network in a Seasonally Dry Landscape
Source: Ecol Evol. 2024 Oct 9;14(10):e70367. doi: 10.1002/ece3.70367 (PMC11461904; doi:10.1002/ece3.70367)
Supplement: Supplementary file 1 — Data S1. Supporting information. [file ECE3-14-e70367-s001.zip › 20240910_SupInfo001_bat-flower network.pdf]

Supporting Information for:

High resource overlap and a consistently generalised pattern of interactions in a bat-flower network in a seasonally dry landscape

Contents

Supplemental Methods 2

DNA extraction 2

PCR amplification and sequencing 2

Reference library 3

Literature cited 4

Supplemental Results 5

Supplemental Figures 6

Figure S1. Rarefaction curves showing sampling completeness of bat-flower interactions. 6

Figure S2. Bipartite interaction networks of plant species visted by nectar-feeding bats in the Sayula Basin, Mexico, across during the dry season months (December, February and April) 7

Figure S3. Bipartite interaction networks of plant species visted by nectar-feeding bats in the Sayula Basin, Mexico, across during the wet season months (June, August and October). 8

Figure S4. Boxplots of seasonal network metrics, showing a) linkage density, bat species richness and plant species richness; and b) d', H2' and niche overlap. 9

Supplemental Tables 10

Table S1. Sequences of the universal primer pairs used 10

Table S2. Spreadsheet with details of herbarium samples and GenBank accession numbers for reference sequences, uploaded separately. 10

Table S3. Spreadsheet with details of initial reference list of potential plant species in network, and further check of the distribution and likely chiropterophily of species in final network, uploaded separately. 10

Table S4. Number of bats captured and samples collected, per species and sampling month; and mean number ± SD of plant species identified in individual samples per bat species and sampling month 11

Table S5. Bat-plant interaction network, uploaded separately. 11

Table S6. Codes for plant species used in Figures 2 and Figures S2-3 12

Supplemental Methods

DNA extraction

The Eppendorf tube containing the pollen sample and cotton swab was first vortexed to dislodge the pollen grains from the swab, then the cotton swab removed and the tubes centrifuged to concentrate the pollen at the bottom of the tube. The ethanol was pipetted out and tubes left to air-dry for 15 min. 250 µl of digestion buffer (10 µl 0.5M EDTA, 0.0017g NaCl, 12.5 µl 1M Tris-HCl, 215 µl ddH_20, 12.5 µl 20% SDS)

and 10 µl Proteinase K (10mg/ml) was then added to the tubes. Samples were left to digest overnight at 55 ºC in a rotating oven. Once the sample was digested, 300 µl of 4 M ammonium acetate was added and the solution was vortexed and left at room temperature for 15 min. The sample was centrifuged at 13,000 rpm for 10 min and the supernatant aspirated into a clean tube. The DNA was then precipitated by adding 1 ml 100% ethanol and centrifuged for 10 min at 13,000 rpm. The supernatant was removed, and the DNA rinsed in 500 µl of 70% ethanol and air- dried for 30 min. DNA samples were then stored in a low TE buffer.

PCR amplification and sequencing

In silico checks of primer-target homology of the trnL, matK and rbcL regions indicated that the taxonomic resolution was low, particularly for species groups likely to be present in the bats' diet such as agaves (Asparagaceae) and cacti (Cactaceae). Additionally, these regions are impractically long (>500bp) for dietary studies (Moorhouse-Gann et al., 2018). We therefore used primer pair UniPlantF and UniPlantR to amplify part of the second internal transcribed spacer of nuclear ribosomal DNA (ITS2), a short region typically of 187-380 base pairs that provides a high taxonomic resolution (Chen et al., 2010; Moorhouse-Gann et al., 2018; Table S1).

PCRs to amplify the target ITS2 region of pollen and faecal samples were carried out in 20 µl reaction volumes, containing: 1 µl (pollen) or 2 µl (faeces) DNA template; 10 µl Qiagen Multiplex PCR Master Mix; 2 µl F primer (at 2 µM); 2 µl R primer (at 2 µM); made up to 20 µl with ddH_2O. Reaction conditions were an initial denaturation step at 95 ºC for 15 min, followed by 34 cycles of 94 ºC for 30 secs, 58 ºC for 30 secs, and 72 ºC for 1 m, and a final extension of 72 ºC for 10 min. The annealing temperature was decided after carrying out a gradient analysis from 50 ºC to 60 ºC on six samples of plant tissue from different families. Successful amplification was determined by visual inspection of a 1% agarose gel stained with ethidium bromide. Each PCR plate

contained two PCR blanks, which were subsequently treated in the same way throughout the library preparation as the samples.

We then performed a second PCR step to add uniquely indexed Illumina adaptors to the amplicons from each sample. All samples were processed in duplicate from the first PCR stage (after DNA extraction) resulting in two PCR replicates of each sample, multiplexed into two separate pools. Each pool contained 260 samples including 8 PCR negatives, and was sequenced on a separate Illumina MiSeq sequencing platform, using 250 bp paired-end reads.

Reference library

First, we conducted a literature review of families of plants known to contain bat-pollinated species (Fleming and Valiente-Banuet, 2002; Stoner et al. 2003; Fleming et al. 2009; Lobova et al. 2009; Avila-Cabadilla et al. 2012) and plant species found in the study region (Macías-Rodríguez, 2004; Sahagún Godínez et al. 2014; Macías Rodríguez et al. 2018). Where no existing literature on pollination syndrome was found for species found in the study region, we assessed flower suitability by comparing flower and plant form to characteristics associated with chiropterophily (flower size, shape and colour; plant biological form).

We then collected 76 tissue samples of plant species from the Herbario “Luz María Villarreal de Puga” (IBUG) and the botanical collection of Cactaceae and Succulents (CUCBA), both in the Department of Botany and Zoology at the University of Guadalajara (Table S2).

We extracted DNA from the plant samples using a modified CTAB method adapted from Särkinen et al. (2012). PCR was carried out in volumes of 20 μl with reagents in the same concentrations as detailed above, with 1 μl DNA template, under the same reaction conditions. Sequences were amplified using primer pair UniPlantF and UniPlantR (Table S1; Moorhouse-Gann et al. 2018). Where these primers failed to generate clean reference sequences, primer pair S2F and S3R were used to amplify the complete ITS2 region and partial 5.8S and 28S sequences (Table S1; Chen et al. 2010). PCR products were sequenced in both directions by an ABI 3730 48-capillary DNA analyzer (Applied Biosystems). Contigs were constructed and consensus sequences generated after editing in BioEdit, and alignment in Mega. In some cases, particularly for members of the Cactaceae, multiple bands of DNA were present on the agarose gel, preventing the generation of clean Sanger sequences. We therefore sequenced 19

plant samples on a 2 x 250bp MiSeq Nano, after following the same library preparation protocol as for the pollen and faecal samples. This has the added benefit of allowing the sequencing of potential multiple ITS polymorphisms within an individual. We successfully generated reference sequences for 55 species, which were then submitted directly to GenBank (Table S2). We failed to generate reference sequences for 21 species, which was likely due to poor yield of extracted DNA.

Literature cited

Avila-Cabadilla, L.D., Sanchez-Azofeifa, G.A., Stoner, K.E., Alvarez-Anorve, M.Y., Quesada, M. and Portillo-Quintero, C.A., 2012. Local and landscape factors determining occurrence of phyllostomid bats in tropical secondary forests. *PloS one*, *7*(4), p.e35228.

Chen, S., Yao, H., Han, J., Liu, C., Song, J., Shi, L., Zhu, Y., Ma, X., Gao, T., Pang, X. and Luo, K. … and Leon, C. 2010. Validation of the ITS2 Region as a Novel DNA Barcode for Identifying Medicinal Plant Species. *PloS ONE*. 5: p. e8613. https://doi.org/10.1371/journal.pone.0008613

Fleming, T. H., and Valiente-Banuet, A. eds. 2002. Columnar cacti and their mutualists: evolution, ecology, and conservation. The University of Arizona Press

Fleming, T. H., Geiselman, C. and Kress, W. J. 2009. The evolution of bat pollination: a phylogenetic perspective. *Annals of Botany* 104: 1017-1043. https://doi.org/10.1093/aob/mcp197

Lobova, T. A., Geiselman, C. K. and Mori, S. A. 2009. Seed dispersal by bats in the neotropics. New York Botanical Garden.

Macías-Rodríguez, M.Á., Frías-Ureña, H.G., Contreras-Rodríguez, S.H. and Frías-Castro, A. 2018. Vascular Plants and Vegetation of the Sayula sub-basin, Jalisco, Mexico. *Taxonomy and Floristics* 96: 103–137. https://doi.org/10.17129/botsci.1030

Macías-Rodríguez, M. A. 2004. *Vegetación y flora de la Laguna de Sayula : guía ilustrada*. 1. Ed. Las Agujas, Zapopan, Jalisco, México : Universidad de Guadalajara.

Moorhouse-Gann, R.J., Dunn, J.C., De Vere, N., Goder, M., Cole, N., Hipperson, H. and Symondson, W.O. 2018. New universal ITS2 primers for high-resolution herbivory analyses using DNA metabarcoding in both tropical and temperate zones. *Scientific Reports* 8: p.8542. https://doi.org/10.1038/s41598-018-26648-2

Sahagún Godínez, E. Macías Rodríguez, M.A., Carrillo Reyes, P., Larrañaga González, N. and Vázquez García, J.A. 2014. Guía de campo de los árboles tropicales de la Barranca del Río Santiago en Jalisco, México. Universidad Autónoma de Guadajara

Särkinen, T., Staats, M., Richardson, J.E., Cowan, R.S. and Bakker, F.T. 2012. How to Open the Treasure Chest? Optimising DNA Extraction from Herbarium Specimens. *PLoS ONE* 7: p. e43808. https://doi.org/10.1371/journal.pone.0043808

Stoner, K.E., -Salazar, K.A.O., Fernández, R.C.R and Quesada, M. 2003. Population dynamics, reproduction, and diet of the lesser long-nosed bat (*Leptonycteris curasoae*) in Jalisco, Mexico: implications for conservation. *Biodiversity & Conservation* 12: 357-373. https://doi.org/10.1023/a:1021963819751.

Supplemental Results

Occurrences of plant species from the Convolvulaceae (129 occurrences), Cleomaceae (104), Malvaceae (91) and Fabaceae (83) families dominated the network, with further occurences from the Capparaceae (59), Acanthaceae (56), Cucurbitaceae (24), Cactaceae (24), Asparagaceae (16), Nyctaginaceae (14), Euphorbiaceae (13), Musaceae (7), Passifloraceae (5), Combretaceae (4), Bignoniaceae (2) and Rubiaceae (2). Cactaceae species were an important resource in February, with a high frequency of occurrence of *Stenocereus queretaroensis, S. dumortieri* and *Pachycereus pecten-aboriginum* (Figure 1). Bat-pollinated canopy trees found in tropical deciduous forest were an important resource throughout the year, including *Ceiba aesculifolia/acuminata*, *Crateva palmeri,* arborescent species in the *Ipomoea* genus, particularly *Ipomoea murucoides,* and *Ruellia bourgaei* (Figure 1).

Supplemental Figures

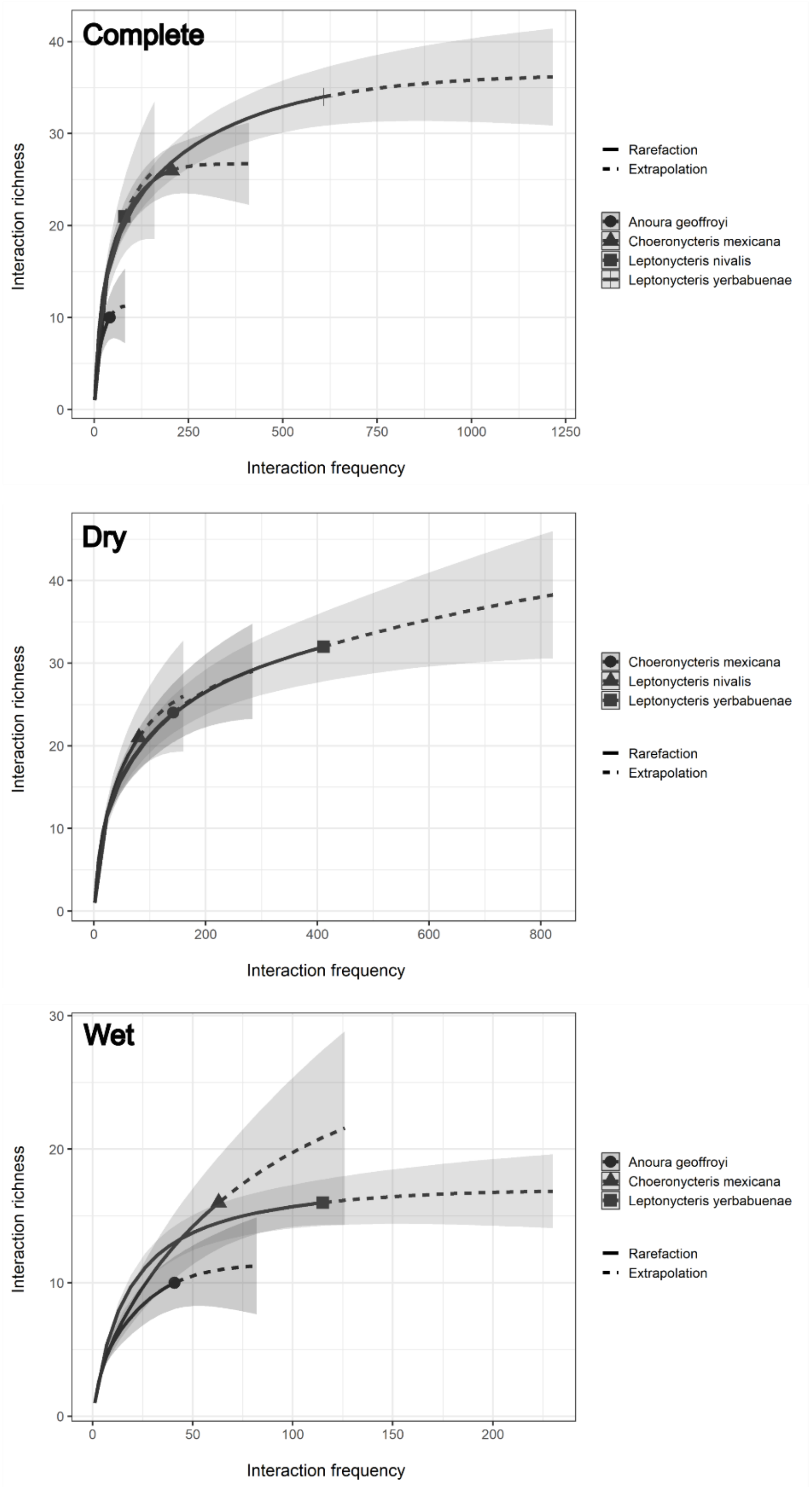

Figure S1. Rarefaction curves showing sampling completeness of bat-flower interactions. Abundance-based rarefaction (solid line segment) and extrapolation (dotted line segments) curves, considering each combination of bat-plant interaction to be a 'species' and their frequency as 'abundances', with 95% confidence intervals (shaded areas). The numbers on the y-axis indicate the observed diversity of bat-flower interactions according to bat species, and the numbers on the x-axis indicate the observed frequency.

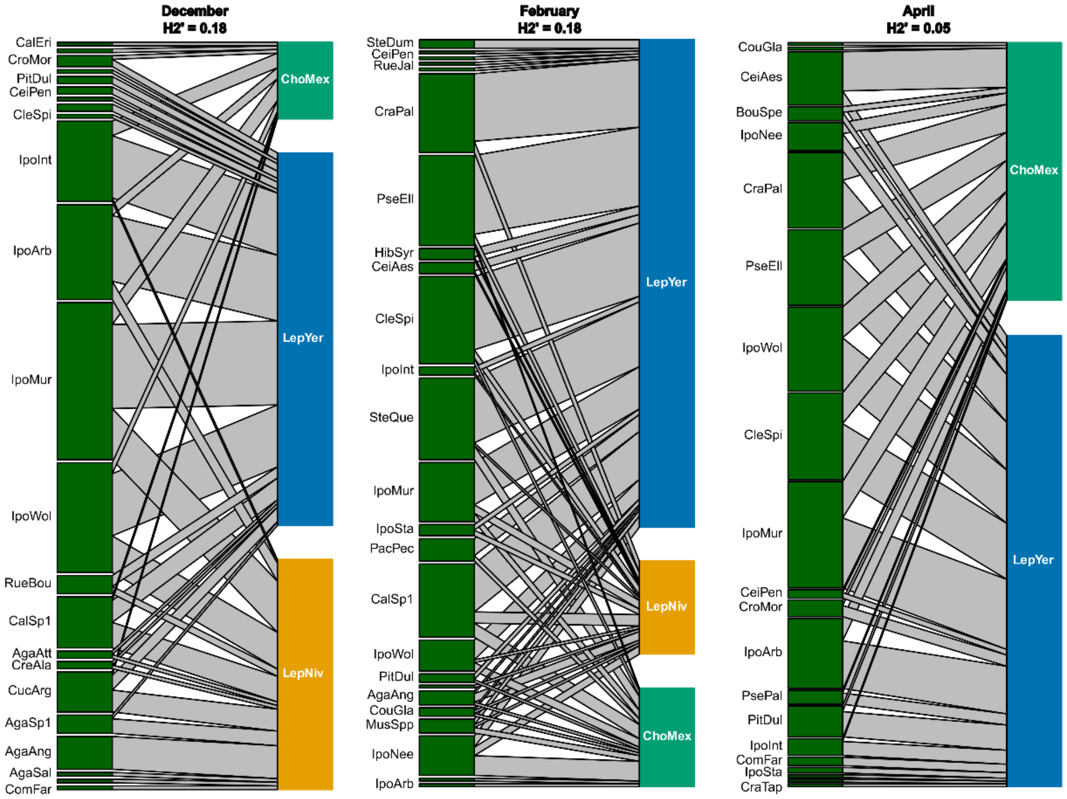

Figure S2. Bipartite interaction networks of plant species visted by nectar-feeding bats in the Sayula Basin, Mexico, across during the dry season months (December, February and April). Bat species labels correspond to: AnoGeo = Anoura geoffroyi, ChoMex = Choeronycteris mexicana, LepNiv = Leptonycteris yerbabuenae, LepYer = Leptonycteris yerbabuenae. Plant species codes are defined in Table S5.

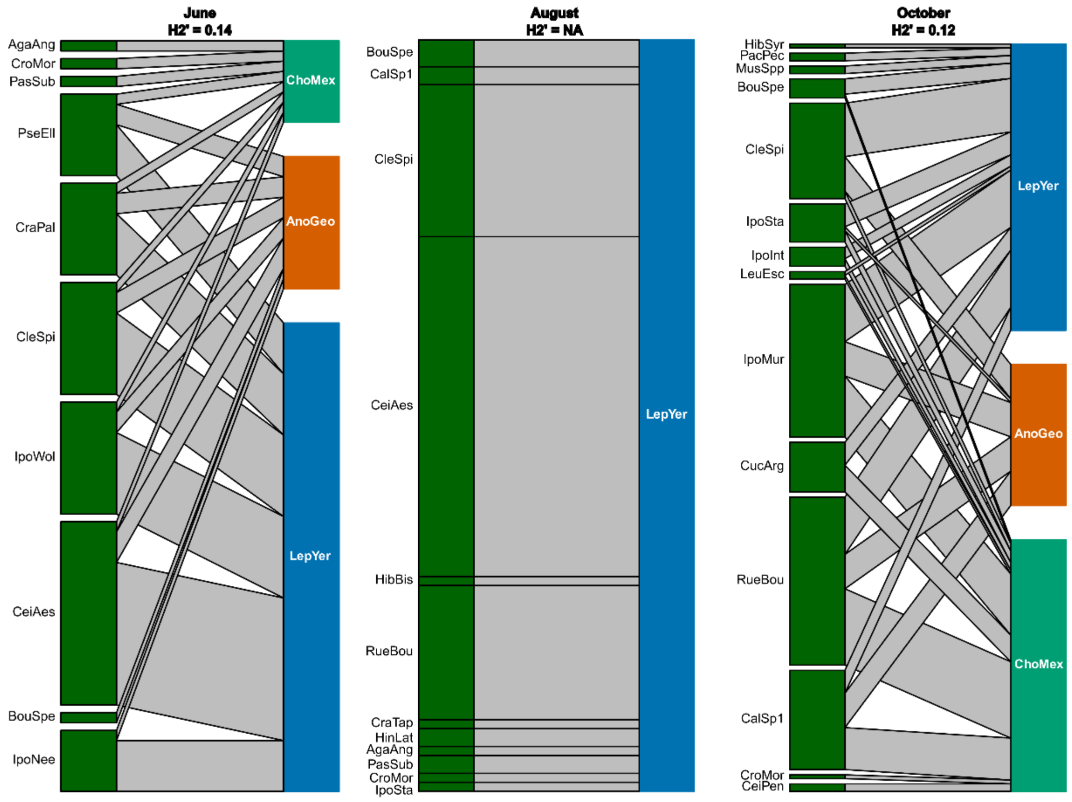

Figure S3. Bipartite interaction networks of plant species visted by nectar-feeding bats in the Sayula Basin, Mexico, across during the wet season months (June, August and October). Bat species labels correspond to: AnoGeo = Anoura geoffroyi, ChoMex = Choeronycteris mexicana, LepNiv = Leptonycteris yerbabuenae, LepYer = Leptonycteris yerbabuenae. Plant species codes are defined in Table S5.

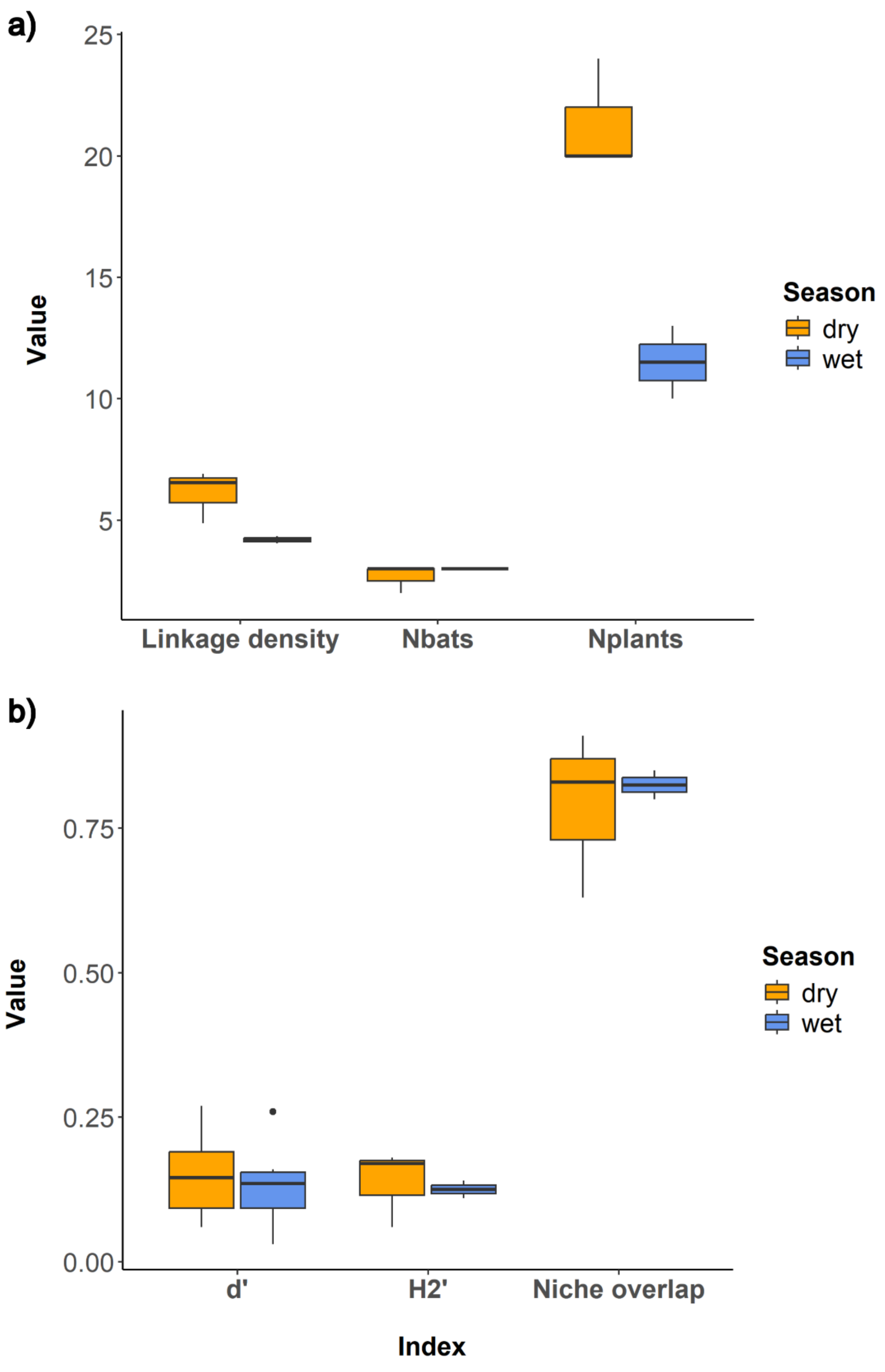

Figure S4. Boxplots of seasonal network metrics, showing a) linkage density, bat species richness and plant species richness; and b) d', H2' and niche overlap, across the dry season months (December, February and April) and the wet season months (June and October).

Supplemental Tables

Table S1. Sequences of the universal primer pairs used.

Name	Region	Sequence 5' – 3'	Reference
UniPlantF	ITS2	TGTGAATTGCARRATYCMG	Moorhouse-Gann et al. 2018
UniplantR	ITS2	CCCGHYTGAYYTGRGGTCDC	
S2-F	ITS2	ATGCGATACTTGGTGTGAAT	Chen et al. 2010
S3-R	ITS2	GACGCTTCTCCAGACTACAAT	

Table S2. Spreadsheet with details of herbarium samples and GenBank accession numbers for reference sequences, uploaded separately.

Table S3. Spreadsheet with details of initial reference list of potential plant species in network, and further check of the distribution and likely chiropterophily of species in final network, uploaded separately.

Table S4. Number of bats captured and samples collected, per species and sampling month; and mean number ± SD of plant species identified in individual samples per bat species and sampling month. L. yer = *Leptonycteris yerbabuenae*; L. niv = *L. nivalis*; C. mex = *Choeronycteris mexicana*; A. geo = *Anoura geoffroyi*

Month		**April**	**June**	**August**	**October**	**December**	**February**
Year				**2017**			**2018**
Season		Dry	Wet	Wet	Wet	Dry	Dry
% bats pollen	*L. yer*	100	31	69	62	87	91
	C. mex	100	25	-	100	71	73
	L. niv	-	-	-	-	100	100
	A. geo	-	13	-	82	-	-
Total # individuals sampled	*L. yer*	19	9	24	12	14	22
	C. mex	11	1	-	15	5	6
	L. niv	-	-	-	-	9	4
	A. geo	-	4	-	9	-	-
Mean # plant species per individual	*L. yer*	7.8 ± 2.8	4.4 ± 1.7	2.9 ± 1.3	4.9 ± 1.3	5.0 ± 1.7	5.9 ± 2.3
	C. mex	7.6 ± 1.6	8.0 ± NA	-	3.1 ± 1.5	3.4 ± 0.9	4.8 ± 1.6
	L. niv	-	-	-	-	4.7 ± 1.3	6.5 ± 3.1
	A. geo	-	4.0 ± 2.6	-	3.1 ± 0.3	-	-

Table S5. Bat-plant interaction network, uploaded separately.

Table S6. Codes for plant species used in Figures 2 and Figures S2-3

Species code	Species name
AgaAng	*Agave angustifolia*
AgaAtt	*Agave attenuata*
AgaSal	*Agave salmiana*
AgaSp1	*Agave sp. 1*
BauSp1	*Bauhinia sp. 1*
BouSpe	*Bougainvillea spectabilis*
CalEri	*Calliandra eriophylla*
CalSp1	*Calliandra sp. 1*
CeiAes	*Ceiba aesculifolia/acuminata*
CeiPen	*Ceiba pentandra*
CleSpi	*Cleome spinosa*
ComFar	*Combretum farinosum*
CouGla	*Coursetia glandulosa*
CraPal	*Crateva palmeri*
CraTap	*Crateva tapia*
CreAla	*Crescentia alata*
CroMor	*Croton morifolius*
CucArg	*Cucurbita argyrosperma*
HibBis	*Hibiscus biseptus*
HibRos	*Hibiscus rosa-sinensis*
HibSyr	*Hibiscus syriacus*
HinLat	*Hintonia latiflora*
IpoArb	*Ipomoea arborescens*
IpoInt	*Ipomoea intrapilosa*
IpoMur	*Ipomoea murucoides*
IpoNee	*Ipomoea neei*
IpoSta	*Ipomoea stans*
IpoWol	*Ipomoea wolcottiana*
LeuEsc	*Leucaena esculenta*
MusSp	*Musa sp.*
PacPec	*Pachycereus pecten-aboriginum*
PasSub	*Passiflora subpeltata*
PitDul	*Pithecellobium dulce*
PseEll	*Pseudobombax ellipticum*
PsePal	*Pseudobombax palmeri*
RueBou	*Ruellia bourgaei*
RueJal	*Ruellia jaliscana*
SteDum	*Stenocereus dumortieri*
SteQue	*Stenocereus queretaroensis*
