# Supplementary material for: High Resource Overlap and a Consistently Generalised Pattern of Interactions in a Bat–Flower Network in a Seasonally Dry Landscape
Source: Ecol Evol. 2024 Oct 9;14(10):e70367. doi: 10.1002/ece3.70367 (PMC11461904; doi:10.1002/ece3.70367)
Supplement: Supplementary file 1 — Data S1. Supporting information. [file ECE3-14-e70367-s001.zip › SUPPORTING INFORMATION.docx]

# SUPPORTING INFORMATION

# Additional supporting information can be found online in the Supporting Information section at the end of this article.

| Filename | Description |
| --- | --- |
| SupInfo001_bat-flower network.pdf | Supplemental Methods  DNA extraction  PCR amplification and sequencing  Reference library  Literature cited  Supplemental Results  Supplemental Figures  Figure S1. Rarefaction curves showing sampling completeness of bat- flower interactions  Figure S2. Bipartite interaction networks of plant species visted by nectar-feeding bats in the Sayula Basin, Mexico, across during the dry season months (December, February and April).  Figure S3. Bipartite interaction networks of plant species visted by nectar-feeding bats in the Sayula Basin, Mexico, across during the wet season months (June, August and October).  Figure S4. Network metrics in the dry and wet season months.  Supplemental Tables  Table S1. Sequences of the universal primer pairs used.  Table S2. Spreadsheet with details of herbarium samples and GenBank accession numbers for reference sequences, uploaded separately.  Table S3. Spreadsheet with details of initial reference list of potential plant species in network, and further check of the distribution and likely chiropterophily of species in final network, uploaded separately.  Table S4. Number of bats captured and samples collected, per species and sampling month; and mean number ± SD of plant species identified in individual samples per bat species and sampling month.  Table S5. Bat-plant interaction network, uploaded separately.  Table S6. Codes for plant species used in Figure 2 and Figures S2-3 |
| SupInfo003_Table S2.xlsx | Spreadsheet with details of herbarium samples and GenBank accession numbers for reference sequences |
| SupInfo003_Table S3.xlsx | Spreadsheet with details of initial reference list of potential plant species in network, and further check of the distribution and likely chiropterophily of species in final network |
| SupInfo004_Table S4.csv | Bat-plant interaction network. |
